# From Nano-Crystals to Periodically Aggregated Assembly in Arylate Polyesters—Continuous Helicoid or Discrete Cross-Hatch Grating?

**DOI:** 10.3390/nano13061016

**Published:** 2023-03-11

**Authors:** Cheng-En Yang, Selvaraj Nagarajan, Widyantari Rahmayanti, Chean-Cheng Su, Eamor M. Woo

**Affiliations:** 1 Department of Chemical Engineering, National Cheng Kung University, No. 1, University Road, Tainan 701-01, Taiwan; 2 Department of Chemical and Materials Engineering, National University of Kaohsiung, No. 700, Kaohsiung University Rd., Nan-Tzu Dist., Kaohsiung 811-48, Taiwan

**Keywords:** nano- to micro-patterns, arylate polymers, self-assembly

## Abstract

This work used several model arylate polymers with the number of methylene segment n = 3, 9, 10, and 12, which all crystallized to display similar types of periodically banded spherulites at various T_c_ and kinetic factors. Universal mechanisms of nano- to microscale crystal-by-crystal self-assembly to final periodic aggregates showing alternate birefringence rings were probed via 3D dissection. The fractured interiors of the birefringent-banded poly(decamethylene terephthalate) (PDT) spherulites at T_c_ = 90 °C revealed multi-shell spheroid bands composed of perpendicularly intersecting lamellae bundles, where each shell (measuring 4 μm) was composed of the interior tangential and radial lamellae, as revealed in the SEM results, and its shell thickness was equal to the optical inter-band spacing (4 μm). The radial-oriented lamellae were at a roughly 90° angle perpendicularly intersecting with the tangential ones; therefore, the top-surface valley band region appeared to be a submerged “U-shape”, where the interior radial lamellae were located directly underneath. Furthermore, the universal self-assembly was proved by collective analyses on the three arylate polymers.

## 1. Introduction

Crystal deformation into variety of geometric shapes is common in nature. Almost a century ago, Ferdinand Bernauer [1] certainly was an early-day pioneer on probing variety of crystal deformation by analyzing more than 400 compounds and reached a conclusion that many of these molecular crystals can be made to grow as twist helices or other deformations that could be conveniently observed under polarized-light optical microscopes. Over the past two decades, in a series of systematic investigations and a comprehensive review, Kahr et al. [2,3,4] probed many representative cases of small-molecule compounds (both organic and inorganic), which are worthy as comparative background information for addressing similar issues in polymer crystals. Long-chain polymeric crystals, other than chain-folding in lamellae, share some interesting common features of crystal deformation as those found in small-molecule compounds. Woo and Lugito [5] further summarized this in a review article demonstrating novel approaches by 3D dissection into interior lamellae with periodic-banded spherulites of many polymers to disclose novel mechanisms with discontinuity in corrugate-board structures, and the review also provided summary evidence for interior periodic assembly matching with optical inter-ring spacing.

Homologous series of arylate polyesters are widely studied. Polymorphic poly(butylene terephthalate) (PBT) reportedly displays crystal transformation [6,7]; yet, it does not form ring-banded aggregates at any T_c_. Polyarylates with longer methylene groups than that of PBT, such as poly(pentamethylene terephthalate) (PPenT), poly(hexamethylene terephthalate) (PHT), and poly(heptamethylene terephthalate) (PHepT), are not as widely studied due to the fewer commercial applications. PPenT can transform from the α form to the β form [8,9], where both the α and β forms are characterized by triclinic chain packing in unit cells, which differ only in the c-axis but remain the same in other lattice parameters. The α-crystal cell is seen in PPenT under zero tension; by contrast, the β-modification dominates if crystallized under high tensions [8,9]. By comparison, PPenT, possessing one more methylene unit than PBT in its repeat unit and, thus, a lower melting point, has been less studied. PPenT has an equilibrium T_m_ value of 149.4 °C, as reported in the literature [8], much lower than that of PBT due to the even–odd effects in the main chains. Regardless of the similar polymorphism behavior of PBT and PPenT, PBT never crystallizes into periodic ring-banded spherulites; by contrast, PPenT displays distinct ring-banded spherulites [10]. PBT is similar to PPenT in that they both display polymorphic crystal lattices, but the former does not pack into periodic banded aggregate upon crystallization, while the latter does. Polymorphic PHT, similar to polymorphic PBT, does not crystallize into periodic ring-banded aggregates [11]; by contrast, PHepT, with one more methylene segment in the repeating unit than that in PHT, readily crystallizes into ring-banded crystals [12]. The even–odd effect superficially appears to work in the formation of ring-banded polymer spherulites; however, it may be too early to predict. Poly(octamethylene terephthalate) (POT) [13], with an even number of methylene segments in the repeat unit, also easily crystallizes to display periodic bands at a suitable T_c_.

A polymorphic polymer can form a ring-banded pattern or a ringless one; conversely, a monomorphic polymer can also form ring-banded aggregates or nonbanded ones. Obviously, the above factual comparison suggests that polymorphism, with more than one crystal lattice form, in polymers is not a determining factor that accounts for the formation of periodic bands or nonbanded ones. The brief survey also indicates that all these arylate polyesters naturally chain-fold upon packing in their long chains into crystalline lamellae of finite thickness (ca. 10–15 nm) and, thus, all inevitably have surface stresses in the lamellae; however, the facts are that some display ring bands but others do not—suggesting that chain-fold stresses might not be a determining factor. From the fact that not all polymers display ring bands, there appears to be a contradiction of the proposition of surface-stress-induced lamellae twist, by applying the classical Aristotle’s proof-by-contradiction. By observing from these experimental facts, chemical structural and kinetic factors both can influence the morphology patterns and periodicity in the final crystallized aggregates of aryl polyesters [9,10,11,12,13,14,15,16]. Recently, poly(nonamethylene terephthalate) (PNT) [14,15,16,17] was found to display not just one single type of ring band but two dramatically different types of ring bands when crystallized at a specific T_c_, suggesting that periodic bands in polymer aggregates cannot be interpreted by the classical continuous helix-twist models. Aryl polyesters may possess polymorphism; however, monomorphism or not in the crystal-unit lattices usually cannot be correlated with the formation of multiple types of spherulites. Monomorphic poly(octamethylene terephthalate) (POT) displays multiple types of spherulites; poly(heptamethylene terephthalate) (PHepT) can possess fractions of α and β crystal lattices and can exhibit many different spherulite patterns with peculiarly varying periodicity [18]. Although commercially available PTT has been widely investigated [19,20,21,22,23,24,25], arylate polyesters and their crystal morphologies have been less probed [20,21,22,23,24,25,26,27,28].

The lamellar assembly of PDT in comparison to a series of other arylate polyester spherulites for more universal proofs was the aim of this work. The governing mechanisms of self-assembly into the intriguing periodically banded patterns were analyzed to understand the universal periodicity commonly seen in arylate polyesters, such as PTT, in comparison to other arylates with a longer methylene segment in repeat units, such as PDT, PNT, POT, and PDoT. The crystal lattice structures of many arylate polyesters might have been classically studied using X-ray [29]; however, the higher-hierarchical lamellar aggregation into periodically crystal assembly has rarely been investigated on these arylate polyesters owing to the complexity of the assembly mechanisms. By following the preliminary and pioneering investigations on periodic assembly of several simple arylate polyesters [26], this work further expounded the mechanisms in fuller and wider detail, and the grating assembly in the nano- to microstructures with suitable cross-bar pitches responsible for photonic iridescence was demonstrated for universal and unprecedented proofs.

## 2. Materials and Methods

The crystal morphologies of several arylate polyesters in homologous series, with their methylene segments varying systematically from short to long, are discussed here. For comparison purposes, a homologous series of arylate polyesters were analyzed and compared. Poly(decamethylene terephthalate) (PDT or P10T, with 10 methylene segments in each repeating unit) was used. The synthesis procedures for PDT were similar to those used earlier in syntheses of a similar polyester of poly(dodecamethylene terephthalate) (PDoT, m = 12) reported earlier [26,30]. All these polyarylates are not commercially available and had to be synthesized in-house with a two-step polymerization, with procedures the same as those earlier described in a previous work. Briefly, for reference, the procedure is restated here: monomers of 1,10-decanediol and dimethyl terephthalate (DMT) with 0.1% butyl titanate as a catalyst were heated in vessels via ester exchanges. The purification of the products was properly conducted by washing out traces of unreacted monomers or impurities. The weight-averaged molecular weight (M_w_) of the synthesized PDT, as determined by gel-permeation chromatography (GPC, Waters), was 21,000 g/mol with PDI = 2.02. Other arylate polyester, poly(dodecamethylene terephthalate) (PDoT, m = 12), was similarly synthesized using respective diols; for brevity, they are not all listed here [26,30]. PDoT (m = 12) has M_n_ = 9224 g mol^−1^ and the polydispersity index (PDI) = 1.91, as determined by gel-permeation chromatography, which has a low glass transition temperature and a medium melting temperature at −1.3 and 121 °C, respectively. Poly(trimethylene terephthalate) (PTT, m = 3) is commercially available Industrial technology research institute, Hsinchu, Taiwan and has a glass transition (*T_g_*) and melting temperature (*T_m_*) of 45 °C and 228 °C [31], respectively.

The film specimens were prepared by drop-casting. The polymers were dissolved in chloroform at ~4 wt.% by stirring. The homogeneous solution was then cast on the glass slide as thin films and dried by placing at 30 °C in a vacuum oven to remove the residual solvent. The film samples were first heated to a suitable maximum melting temperature (T_max_) for 2 min on top of a hotplate for erasing the thermal history and then removed rapidly from the hotplate to a temperature-controlled hot stage (temperature precision ± 0.5 °C) being preset at the intended crystallization temperatures till full crystallization. For the interior dissection of the morphology, fracturing on the specimens (thickness 10–20 μm) was performed. The films of the crystallized samples (at a controlled film thickness and T_c_) on the glass substrates were fractured by precutting the glass substrates to direct the intended fracture propagation. The fractured pieces of the samples were affixed onto metal stands at proper orientation angles using silver-tape glues at suitable inclination angles with respect to the electron beams in the SEM vacuum chamber, Tokyo, Japan.

### Apparatus and Procedures

A polarized-light optical microscope (POM, Nikon Optiphot-2, Tokyo, Japan) equipped with a Nikon Digital Sight (DS-U1) digital camera and a microscopic heating stage (Linkam THMS-600 with T95 temperature programmer, Linkam Scientific Instrument Ltd., Surrey, UK) was used.

Atomic-force microscopy (AFM diCaliber, Veeco Corp., Santa Barbara, CA, USA) investigations on the top surfaces of the cast film samples were made in an intermittent tapping mode with a silicon tip (f_0_ = 70 kHz, r = 10 nm) installed. The largest scan range was 150 μm × 150 μm, and the scan was kept at 0.4 Hz for the overview scan and zoomed-in regions.

High-resolution field-emission scanning electron microscopy (Hitachi SU8010, HR-FESEM, Tokyo, Japan) was used for revealing the interior lamellar assembly in the exposed fracture surfaces/interiors, which was to be correlated with the morphology and ring patterns on the top free surfaces. The samples, after fracturing and setting on metal stands, were coated with gold or platinum vapor deposition using vacuum sputtering prior to the SEM characterization.

## 3. Results and Discussion

### 3.1. Interior Crystal Assembly in Birefringence-Banded PDT Spherulite

**Figure 1:**
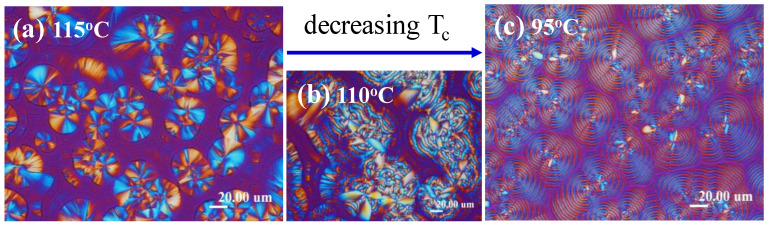
POM micrographs of PDT melt crystallized at decreasing T_c_: (**a**) 115 °C; (**b**) 110 °C; (**c**) 90 °C, held till full crystallization by quenching from T_max_ = 165 °C. Reprinted with permission from ref. [32]. Copyright 2021 Elsevier.

The crystallization temperature (T_c_) kinetically influences the mechanisms of nucleation and growth. For PDT, two dramatically different types of ring-banded patterns were present at high versus low T_c_ [30,32]. For appreciating the difference between these two types of ring bands formed at medium T_c_ (90–95 °C) versus high T_c_ (120–125 °C), Figure 1 demonstrates two dramatically different banding patterns of PDT spherulites, with the film thickness being kept at constant 15–20 μm. Figure 1a illustrates the single extinction-band pattern crystallized at relatively high T_c_ = 115 °C or above, while interestingly, Figure 1b shows an intermediate pattern between these two types at an intermediate T_c_ = 110 °C, which was composed of “dual-ring bands” in the central core but extinction bands at the outer rims with a unique core–shell pattern. That is, the spherulitic aggregate was actually a composite core–shell morphology of two discrete types of optical birefringence patterns. Figure 1c shows dual-birefringence ring bands crystallized at T_c_ = 90–95 °C or lower. The mechanisms of the crystal assembly in the dual-birefringence ring-banded PDT at T_c_ = 90 °C of thicker films were therefore different from that in the epicycloid-extinction PDT at higher T_c_ = 110–125 °C and in thinner films. As discussed in an earlier work [32], another type of ring band, termed epicycloid-extinction-banded PDT spherulites, at higher T_c_ = 110–125 °C is sensitively dependent on the film thickness, with the epicycloid-extinction band patterns disappearing completely at film thickness >10 μm, and the inter-band spacing increasing dramatically with respect to the increasing film thickness. Note that the inter-band spacing of the PDT spherulite was 3–5 μm at T_c_ = 95 °C (Figure 1c), which means that the film thickness should not be lower than 3–5 μm if dual-birefringence bands are to be packed in the PDT specimens. When the film thickness was lower than this critical value (inter-band spacing), then no dual-birefringence bands were observed in the crystallized films; instead, only bands with an optical extinction border (termed “extinction band”) were present. This is easy to understand, as thin films constrain lamellae to be normal oriented in cross-hatch patterns.

Thus, in dramatic contrast to the extinction-banded spherulites (with successive bands bordered with a sharp extinction ring) crystallized at high T_c_ (120 °C or above), the crystallization of the same PDT films at low to intermediate T_c_, such as T_c_ = 90–95 °C, led to optically dual-birefringent spherulites of distinct alternating blue/orange rings in the spherulites, where the ring bands were not bordered with optical extinction but were featured with alternate birefringence colors in POM with tint plates. This behavior of no dual-birefringence bands in PDT at T_c_ = 90 °C was opposite to that of PDT at higher T_c_ = 120 °C, where only extinction bands could be present, as discussed. This fact suggests that the nature of the extinction PDT bands might be significantly different from that in the dual-birefringence PDT bands. Although both extinction bands and dual-birefringent bands were featured with distinct periodicity in the circular rings, the dramatic differences in the optical birefringence properties in these two types of PDT spherulites suggest that the interior crystal-lamellae assembly may significantly differ with respect to their respective optical birefringence patterns. The effect of the film thickness (3–20 μm) did not appear to influence the morphological patterns of the birefringent spherulites with blue/orange bands. Note that for thick PDT films with too high optical retardation (τ), the optical light does not penetrate easily; thus, the blue/orange birefringence could not be contrasted in visible patterns. However, the birefringent ring bands of PDT spherulites at T_c_ = 90 °C remained similar in pattern and displayed same inter-band spacing (ca. 3–4 μm) for PDT film samples, which did not change much with the increase in the film thickness (from ca. 5–20 μm).

Not only T_c_ but also confinement by film thickness might influence the lamellar assembly and, thus, birefringence patterns, as seen in POM. A preliminary investigation revealed that a minimum film thickness (3–5 μm) of PDT specimens was required to display dual-birefringence ring bands. Supplementary Materials Figure S1 shows POM graphs of PDT spherulites of four different levels of film thickness: (a) ultra-thin at 300~500 nm, (b) 3~5 μm, (c) 8~10 μm, and (d) 15~20 μm, all at T_c_ = 90 °C. The very thin PDT film (ca. 300–500 nm) did not exhibit any discernible dual-birefringence ring bands upon crystallization at T_c_ = 90 °C. The PDT films of all other higher thickness levels (5–20 μm) displayed optically similar dual-birefringent alternating blue/orange bands, with similar inter-band spacing. The effects of the variation of the melt-exposure time (from 1–120 min) at T_max_ = 165 °C on the birefringent PDT spherulites were also investigated. For comparison, Supplementary Materials ESI Figure S2 shows POM graphs for the PDT films (thickness kept constant at 2–3 µm) all melt crystallized at T_c_ = 95 °C with different times (t_max_) held at T_max_ for melting to erase the prior thermal histories: (a) 1 min, (b) 15 min, (c) 30 min, (d) 60 min, (e) 90 min, and (f) 120 min. The results show that all PDT birefringent spherulites remained the same or similar with alternate blue/orange bands, with the same inter-band spacing (4 μm). All spherulites remained similar in size with a radius = 30~50 μm. This fact suggests that the melt/thermal exposure at T_max_ = 165 °C had no effect of altering the birefringent bands and that exposure at this temperature caused no degradation. In addition, it should also be commented here that the birefringent spherulites were not influenced by the top-cover confinement on the films during the crystallization at T_c_ = 95 °C; by contrast, the crystallization of the PDT at T_c_ = 120 °C with top-cover confinement led to no periodic bands (i.e., ringless), yet extinction bands were visibly present if no top-cover was placed on the PDT films.

**Figure 2:**
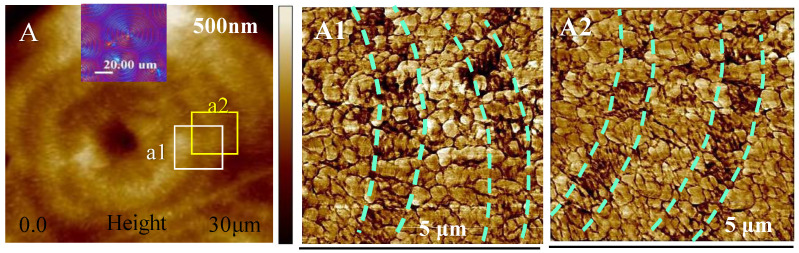
Nanoassembly on the top-surface pattern as viewed in (**A**) AFM height images and (**A1**,**A2**) zoomed-in images of the top surface of the alternate birefringent blue/orange bands in the PDT spherulites at T_c_ = 90 °C (film thickness = 3–5 μm).

Prior to the SEM analysis of the interior lamellae assembly, AFM analysis was performed to reveal the nanopatterns on the top surface of 90 °C crystallized PDT spherulites that displayed dual-birefringent colors with blue/orange bands. Figure 2 shows AFM images of the PDT samples (films of thickness ca. 3–5 μm) crystallized at T_c_ = 90 °C. The ring bands with the alternate optical birefringence (blue/orange) is shown as an inset in Figure 2A. Note that the pattern of the PDT dual-birefringent PDT spherulites (at T_c_ = 90 °C) differed completely from those for the extinction-banded PDT spherulites in thin films at T_c_ = 120 °C, earlier disclosed in a concurrent work on PDT [32]. The zoomed in AFM analysis on the top surface (Figure 2A1,A2) shows a nanograiny feature with alternating low and high bands. The top surface of the 90 °C crystallized birefringent-banded PDT spherulite was composed of apparently grainy polycrystals. Such dual-morphology ring bands are dramatically in contrast to the single-crystalline terrace-like packing on the top surfaces of the 120 °C crystallized PDT extinction-banded spherulite. All these features further reinforce that the nature and mechanisms of the crystals building the birefringent spherulites of the alternating blue/orange bands crystallized at T_c_ = 95 °C or lower should differ widely from those governing the extinction-banded PDT spherulites crystallized at T_c_ = 120 °C or higher. However, the detailed lamellae assembly in the interior of the banded spherulites could still not be discerned from the POM patterns or the AFM analysis on the top surface. The circular dot-like grains on the top surfaces of the PDT dual-birefringence bands suggest that they might be the individual terminal ends of the interior lamellae, as they emerge from the inner bulk to top surface. Note also that the darker bands in Figure 2A1,A2 represent the valley, where the grainy dots are oriented in a different direction in contrast to the elongated crystals in the neighboring brighter bands (ridges). This further suggests that the interior lamellae emerge to the top surface in correspondingly different orientations depending on the valley or ridge bands. A later section unveils the interior crystal assembly responsible for such dual-birefringence PDT bands. Interior dissection into the assembly of the interior lamellae crystals in the birefringent PDT spherulites (crystallized at T_c_ = 90 °C) may shed new light on answering these critical questions. From the AFM phase images, the inter-band spacing = 3–4 μm.

**Figure 3:**
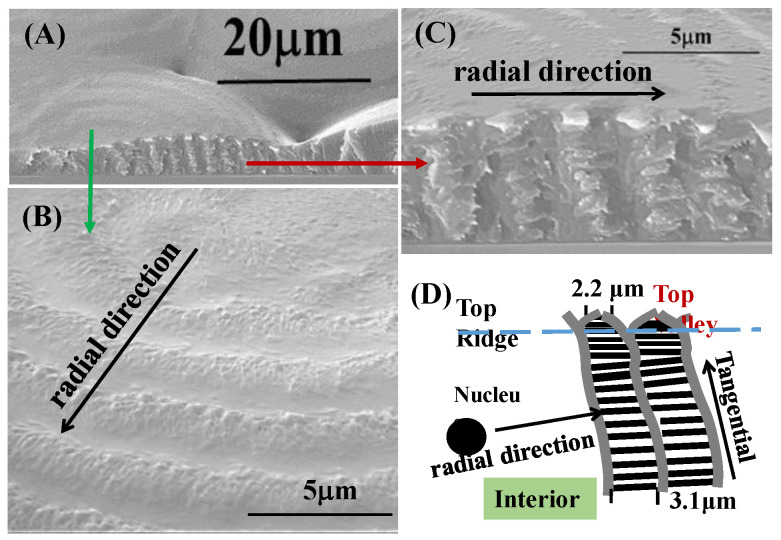
SEM micrographs of the PDT melt crystallized at 90 °C: (**A**) entire fractured specimen; (**B**) top surface; (**C**) fractured surface; (**D**) schemes of shells composed of tangential/radial lamellae alterations assembled as a cross-hatch grating structure (film thickness = ca. 5–10 μm varying from nucleus to periphery).

The band patterns on the top surfaces of the blue/orange birefringent PDT spherulites (at T_c_ = 90 °C) could mislead in the interpretation of the mechanisms of periodic banding if the interior lamellae were not analyzed. Interior dissection by examining the fractured PDT spherulites was performed by SEM characterization. Figure 3A,B show SEM micrographs of the PDT melt crystallized at 90 °C, and Figure 3C,D show schemes illustrating alternate tangential-to-radial lamellae with discontinuity. The PDT films were kept at thickness = ca. 15 μm. All PDT samples were first melted at T_max_ = 165 °C (1 min), then quenched to T_c_ = 90 °C, and held till full crystallization. Both the top surfaces and interior fractured surfaces of the crystallized PDT specimens were examined. The SEM graphs for the top surfaces revealed similar grainy crystal aggregates on the “ridge region”, which is similar to the AFM images discussed earlier. The valley bands on the top surface were of a flat and lower region in comparison to the ridge bands. Apparently, lamellae assembly is not possible by simply examining the top surfaces using either SEM or AFM analysis. As the nucleus center of the spherulite is located near the top surface, the onion-like interior morphology appears like a multi-shell concave-up bowl (i.e., a hemispheroid). The fracture–dissection SEM results in Figure 3B also reveal very critical pieces of evidence showing that the ridge bands on the top surfaces actually correspond to regions where the interior tangential lamellae emerge to the top surfaces; by contrast, the valley bands on the top surfaces correspond to where radial lamellae evolving or bending from the tangential ones. Both the top surface and interior grating-like array clearly reveal that the inter-band spacing was consistently ca. ~3 μm. From the above SEM results for the interiors of the banded PDT (T_c_ = 90 °C), the correlations between the top periodic bands vs. the interior lamellae assembly can be feasibly constructed. The schemes in Figure 3C show the interior lamellae assembled as a mutually intersecting grating. Apparently, the interior lamellae, as revealed in the SEM result for the interior of the banded PDT, are assembled as a cross-hatch grating structure, whose cross-bar pitch = 3 μm and equal to the optical inter-band spacing in the POM images. Except for the interfacial layer, the radial lamellae were always roughly a 90° angle perpendicularly intersecting with the tangential ones; therefore, the top-surface valley band region appeared to be a submerged “U-shape”, where the interior radial lamellae were located directly underneath. In addition, according to the SEM results, the interior tangential lamellae (or their bundles) were connected to the top-ridge region, while the interior radial lamellae were situated underneath the valley region of the top surface. Upon POM characterization with tint plates, if the interior tangential lamellae (crystals oriented in the perpendicular direction) have an orange color, then the interior radial lamellae (crystals oriented mostly in the horizontal direction) have a blue color. The periodicity repeated to produce optical patterns of orange/blue color rings according to the lamellae’s mutual cross-hatching intersections, as shown in Figure 3D.

**Figure 4:**
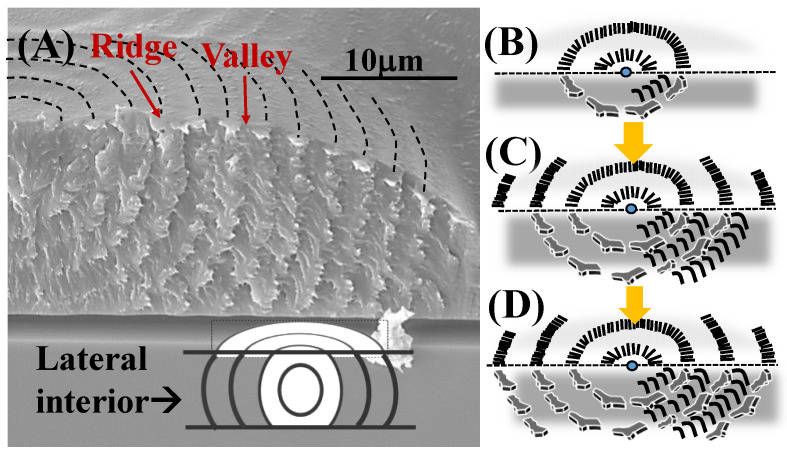
(**A**) SEM graphs of the interiors of the double-ring-banded PDT spherulites packed with hemi-spheroid shells in the tangent circumferential direction interfacing with radial-oriented thinner lamellae; (**B**–**D**) schemes exemplifying the initial growth near the nuclei center eventually to final multiple-packed shells with the interior perpendicularly intersected lamellae.

The dual-birefringence banded PDT spherulites (T_c_ = 90 °C) can be nucleated on the top surface or interiors of films. Figure 4 shows SEM micrographs of fractured cross-section of top-initiated banded PDT spherulites displaying a distinct layered corrugate-board structure. Of the topology, the interior lamellae with corrugate-board (i.e., multi-shell) assembly reached upward to the top surface to form the periodic banding of inter-band spacing ca. 4 μm that exactly matched with the optical spacing. Each of the interior tangential lamellar bundles corresponded to the “ridge region” of the top band patterns. Finer branching lamellae grew roughly perpendicular to the tangential lamellae; thus, these branching lamellae were aligned in the radial directions. The tangential lamellae in the 3D growth were aligned as multi-shelled hemispheroids of increasing radii, which can be clearly seen in Figure 4A. The interior lamellae of hemispheroid geometry, as revealed in the fractured PDT spherulites, can also be viewed as an “onion-like” structure cut into halves, as illustrated in the inset on the bottom of Figure 4A. The SEM graphs also show that the inter-shell distance (~4–5 μm) equaled exactly the inter-ring spacing (~4–5 μm) in the POM graphs for the PDT at T_c_ = 90 °C. The perfect match between the morphology and optical birefringence evidence suggests the validity of the proposed assembly mechanism, leading to the final aggregate’s banding periodicity. These series of schemes for step-by-step growth in Figure 4B–D illustrate three stages of growth: from initiation of nuclei on or near the top surface to complete 3D growth to form a corrugate-board architecture with a hemispheroid geometry. The 90° angle intersection of two species of lamellae accurately accounts for the periodic optical blue–orange birefringence colors, as illustrated. With the nucleus center on the top surface of the thick PDT films, the alternately concentric shells all took a hemispheroid shape. Regardless of the film thickness or location of the nucleus centers, the analyses yielded consistent results that the interiors of the birefringent-banded PDT spherulites (T_c_ = 90 °C) were filled with alternate cross-hatch lamellae mutually intersecting at an oblique or nearly perpendicular angle, where the interior tangential lamellae correspond to the ridge region and interior radial lamellae to the valley region on the top surface.

### 3.2. Mechanisms of Lamellae Packing into Birefringent Bands

**Figure 5:**
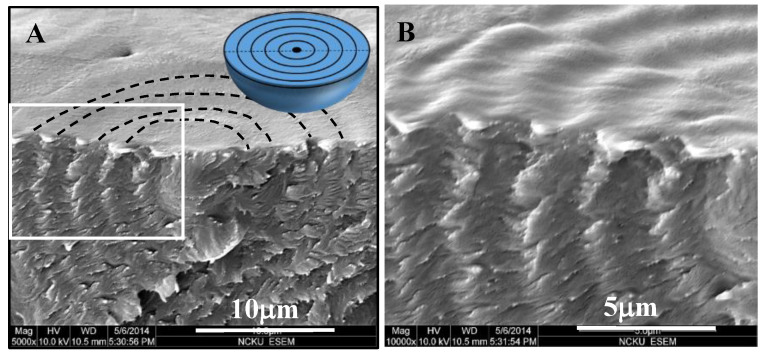
(**A**) SEM micrographs of fractured interior at T_c_ = 90 °C; (**B**) lamellar assembly in the interior of the banded PDT as a cross-hatch grating structure.

Depending on the PDT film thickness and location of nuclei (top, center, or bottom of the film thickness), the fractured interiors revealed correspondingly different geometries of the alternating shell structures. Figure 5 displays SEM micrographs for fractured PDT films of various thickness. Figure 5A shows the PDT film with a thickness of 7 μm, where five bands were present in one spherulite. As a result of the nucleation on the top surface and the constraint of the narrow film thickness, the 3D-banded spherulite took a shape of a concave-up hemispheroid, and the alternating shells curved up as an “arc” shape. By contrast, for the PDT film ~40 μm thick with the nucleus center near the middle zone of a film, Figure 5B shows five alternate and concentric shells in the banded spherulite that took a spheroid shape. In the SEM evidence for the interior of the birefringent-banded PDT spherulites, it is clear that no interior lamellae underwent continuous helix-twisting, such as DNA’s double helices, all originating from a common center. Instead, each of the hemispheroid shells in the banded PDT spherulite was interfaced with discontinuous tangential-to-radial interfaces. Note that some of the tangential-oriented lamellae may branch out or occasionally bend and twist at a ca. 90° angle to merge with the radial lamellae; but such a twist was abrupt at the interfacial regions. If morphological analyses were aimed only on the top surfaces of thin-film specimens, the essential assembly in the 3D-bulk interiors might have been overlooked. It is easy to mistake the occasional branching/twisting on the top surfaces as continuous screw-like helices, when actually there were discontinuous interfaces between the interior crystals that were packed to display successive bands.

### 3.3. Top-Surface Morphology Versus Interior Lamellar Assembly

**Figure 6:**
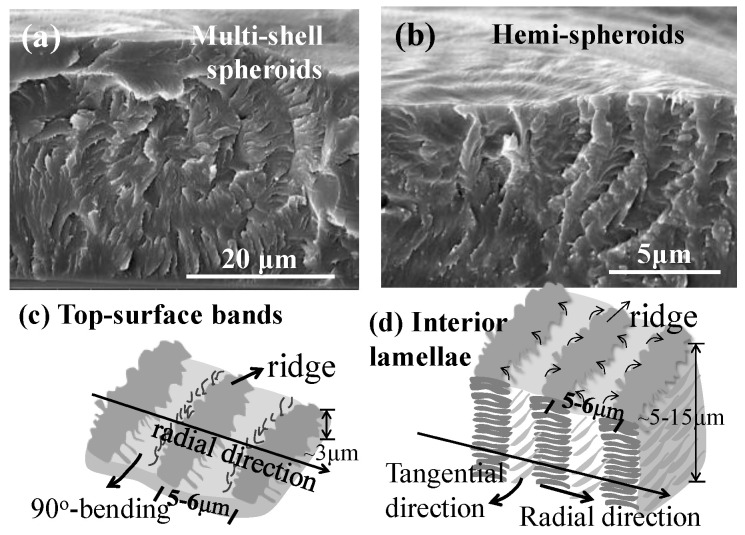
SEM micrographs of the interiors of two different banded PDT spherulites both melt crystallized at 90 °C: (**a**,**b**) nuclei near top surface–multi-shell hemispheroids; (**c**,**d**) schemes for the optical birefringent patterns and top vs. interior assembly (sample film thickness = ca. 20–30 μm).

The fracture of samples might randomly cut across various sections of a banded spherulite, which might yield slightly different patterns of assembly. For proof of the universality of the alternating tangential/radial structures in the banded PDT spherulites, alternative fractured interior surfaces were further examined. Figure 6a,b display SEM graphs for the fractured interiors of birefringent-banded PDT spherulites (all at T_c_ = 90 °C), which clearly revealed a common multi-shelled hemispheroid or spheroid structure (depending on the location of the nucleation sites being near the top surface or middle of the films). In the interior of the birefringent-banded PDT spherulites, the tangential lamellae curved into a bowl shape (i.e., hemispheroid). The interior tangential lamellae were connected to the “ridge region” on the top surface. From the tangential lamellae, some lamellae either flip-twisted at a 90° angle or branch evolved in a perpendicular direction to fill the space between two neighboring tangential shells. The schemes in Figure 6c,d illustrate that the curved tangential shells were attached with a 90° angle bending/twisting or perpendicular branching. The perpendicularly twisted or branched lamellae generally oriented their long axes toward the radial direction of the spherulite. Thus, there were alternative tangential/radial lamellae layers, with each layer being shaped as multi-shelled hemispheroids (i.e., when the curved spheroids are flattened, they become “corrugate-boards”). The radial-oriented lamellae in the banded aggregates were not flat but shaped generally as a concave-up U-shape, forming a valley. From the scheme, the dark wide stripes indicated the “valley region” optical patterns that apparently were situated on top of the interior radial-oriented lamellar plates. The thin, narrow, and solid lines on the top of the scheme indicate the “ridge region” on the top surfaces, which correspond to the protruded spots of the interior tangential-oriented lamellae. The ridge bands on the top surfaces actually correspond to regions where the interior tangential-oriented lamellae emerged to the top surfaces. By contrast, the valley bands on the top surfaces correspond to where the radial lamellae evolved or bent from the tangential-oriented ones.

Apparently, if the analysis was confined to thin films without 3D inner views, investigators might have been misled by the lamellae’s superficial assembly on the top surface, while the majority bulk of the assembly in the 3D interiors would have remained hidden. On the top surface of the thin film specimens, investigators might observe some occasional twisting and bending of lamellae from ridge to valley band. However, the interior lamellae (accounting for 8–9 μm of the bulk), accounting for the majority 90% of the entire bulk, would be buried and skipped. The schematic shows how the interior lamellae might emerge and twist while going to the top surface, wherein the interiors were actually composed of discontinuous shells of crystal plates and branches mutually oriented at some oblique angles.

It has been proved that no continuous helix-twist of single-crystal lamellae is present in the extinction-banded PDT spherulites (crystallized at high T_c_ = 110–120 °C) [30]. For the birefringent (blue/orange)-banded PDT spherulites crystallized at lower T_c_ = 90 °C, the interiors were filled with multi-shell concentric spheroids composed of alternating tangential lamellae that periodically 90° angle twisted and/or branched out to form the radial lamellae. During growth, the tangential lamellae first evolved initially from the sheaf-like nucleus center, which subsequently produced branches in perpendicular orientations to fill the expanding space as they grew outward from the nucleus center to the periphery. Toward growth termination, the periphery of the increasingly larger spherulites started to impinge on neighboring spherulites, where the growth terminated. Eventually, a multi-shell hemisphere aggregate formed in the interiors; on the top surface of the films, concentric multi-bands of a fixed inter-band spacing formed. Furthermore, doubly-birefringent banded PDT spherulites were always filled progressively with periodic branching during growth by starting from quasi-single crystals (lamellar sheaf-bundles) at the nucleus center to a final complex hierarchical aggregations of multiple lamellae. Again, in the architecture of the birefringent PDT spherulites (T_c_ = 90 °C), no continuous helix-twist of the single crystal lamellae was evident, although a sharp 90° twist from the tangential- to radial-oriented lamellae bordering at the discontinuous interfaces was seen in each of the hemi-spheroid shells.

Altogether, the tangential-oriented lamellae and periodically-spawned branches filled the expanding spherulite’s space during growth, which increased with the increasing radius as cubic R (i.e., R^3^). As the tangentially oriented lamellae emerged to the top surfaces of the spherulites, they protruded upward to become the “ridge band”, while the interior radially oriented lamellae, being branches themselves growing at 90° angle from the tangential ones, remained to be submerged but curved up to form a “U-shape” valley band with a flat/smooth texture on the top surface. Furthermore, the tangential-oriented lamellae accounted for the optically blue birefringent ring, while the radial lamellae accounted for the orange rings, as viewed in the POM graphs; vice versa, in the neighboring next quadrant of the POM graphs, the opposite was true.

### 3.4. Universal Features of 3D Interior Assembly in Arylate Polyesters

**Figure 7:**
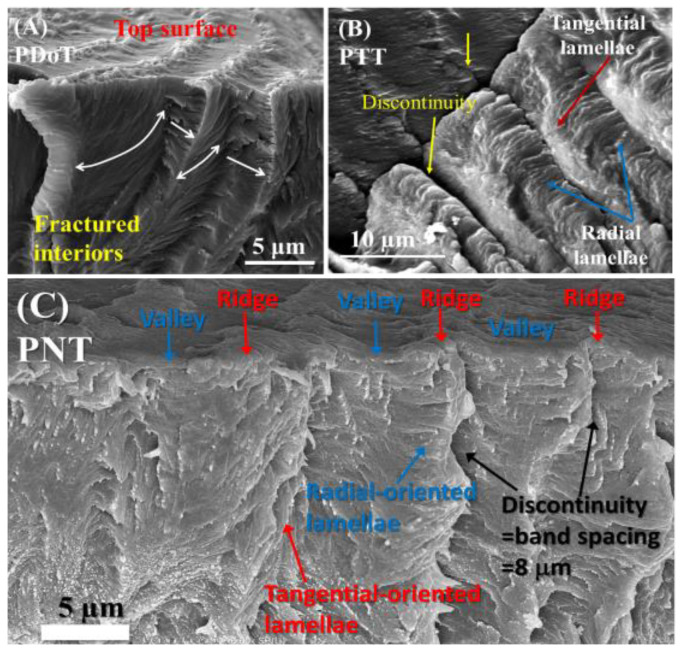
SEM micrographs for comparisons of the assembly analogy of interior lamellae for three arylates: (**A**) PDoT (P12T)-banded spherulite melt crystallized at T_c_ = 96 °C, with shell thickness: 4 μm (same as optical inter-band spacing); (**B**) PTT (P3T)-banded spherulite melt crystallized at T_c_ = 165 °C, with shell thickness = 10 μm (=optical inter-band spacing) [31]; (**C**) type-1 ring band of PNT (P9T) crystallized at T_c_ = 85 °C, with shelled thickness = 8 μm (cross-bar pitch = optical inter-band spacing). (Figure 7B Reprinted with permission from ref. [31]. Copyright 2017 American Chemical Society ).

For the universal comparison of a series of homologous arylate polyesters with PDT (m = 10), the other polyesters (m = 3, 9, 12) were similarly analyzed and compared [19]. Figure 7 shows the dissected morphology results for the interior assemblies in (A) PDoT (m = 12), (B) PTT (m = 3), and (C) PNT (m = 9), respectively. All specimens of the three arylate polyesters were crystallized as bulks at T_c_ = 96, 165, and 85 °C, respectively, to develop distinct ring bands (double birefringence). These three (PDoT, PTT and PNT) were fractured in similar ways as PDT (P10T), sputter-coated with gold, and characterized using SEM. Note that all arylate specimens (PDoT, PTT, PNT) for comparative purposes were recharacterized using the same SEM techniques in this work, although similar interior morphologies for them have been earlier reported in the literature [15,33,34]. One can see that these three arylate polyesters all displayed similar cross-hatch architectures (tangential-radial lamellae perpendicularly intersecting at 90° angle), differing only in the inter-layer shell thickness: 4 μm for PDoT, 10 μm for PTT, and 8 μm for PNT Type-1 band, in comparison to the same cross-hatch structure of PDT with an inter-layer shell thickness of ~5 μm in this work. Note here that PNT is more complex in the banding architecture, as it displays not just one but two entirely different types of ring bands (labeled as Type 1 and Type 2, respectively) [15]; for comparison, only Type-1 PNT was used here. All four arylate polyesters displayed similar interior architectures of cross-hatch tangential/radial intersections, proving the universality of the proposed model being fit with all the ring-banded arylate polyesters investigated here. For most arylate polyesters (from PTT to PDT) crystallized at respectively suitable temperature ranges, their banded assemblies all displayed similar periodic birefringence patterns in the interior crystal assemblies, differing mainly in the inter-band spacing and some trivial details of the assemblies.

**Figure 8:**
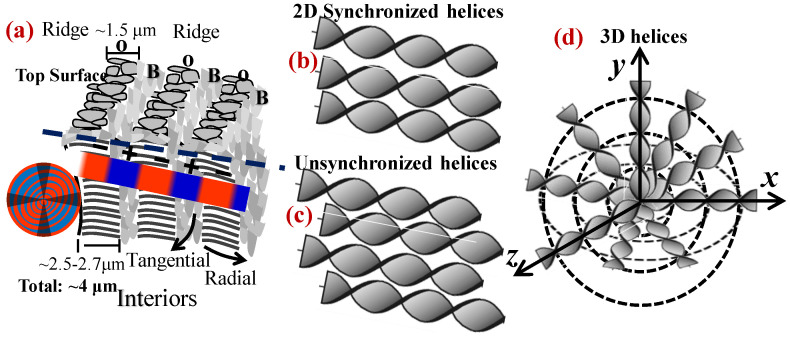
Crystal nanoassemblies of lamellae to higher hierarchical aggregates with the periodicity of PDT: (**a**) 3D cross-hatch with shell layers showing blue/orange birefringence alternate rings; (**b**) 2D helices in perfect synchronized pace showing birefringence/extinction alternate rings; (**c**) 2D helices in disordered pace showing complete optical extinction; (**d**) 3D helices from a common nucleus center. O–orange ring; B–blue rings. Reprinted with permission from ref. [30]. Copyright 2018 Royal Society of Chemistry).

The 3D assembly of PDoT has been analyzed in an earlier work [30]. Polymeric spherulites are 3D aggregates of complex lamellar architectures with periodic branching, sporadic bending, twisting, or scrolling; thus, the interior assembly from inner lamellae to top-surface morphology should not be overlooked in investigating full mechanisms. Figure 8 illustrates three possible assemblies of lamellae in the aggregates of PDT into periodic bands. Figure 8a shows the proposition that the polymeric spherulites are 3D aggregates composed of multi-shells with cross-hatch grating architectures, with complex lamellae of periodic branching and sporadic bending. In this grating architecture, all tangential lamellae are aligned in the same direction and sandwiched in the interfacial layers of two radial-lamellar shells; similarly, the radial-oriented lamellae in the shells are all aligned in the “radial direction”. Thus, the tangential-oriented lamellae display a certain birefringence pattern differing from that of the radial-oriented lamellae and collectively displaying alternate double-ring-banded pattern optically in polarized light. Subsequently, another proposition of continuous helix-twist lamellae is checked and proved by contradiction. Figure 8b shows that if all helix-twist lamellae were synchronized in helix pace, then they would have displayed alternate birefringence-to-extinction bands—an expected phenomenon that certainly contradicts with the experimental proofs according to the POM patterns with the dual-birefringence rings (i.e., both valley and ridge bands are packed with crystals but of different orientations). Finally, in a situation where the synchronized pace of these helix-twist screws is not warranted, then the total optical extinction is a result, which again contradicts with the experiment-observed optical patterns. Figure 8c shows a scheme where if all helix-twist lamellae are not in a synchronized pace but offset a fraction of the pitch from one to another in nonsynchronized alignment, then full optical extinction is the result. This of course would oppose with the POM experimental results for PDT at T_c_ = 90 °C.

By philosophical articulation for verifying these mutually opposite propositions (shown in Graphs-a–d in Figure 8), the classical Aristotle’s proof-by-contradiction for examining the propositions of nanoassembly leading to final hierarchical periodicity was then utilized to testify these propositions by summarizing the results of these three arylate polymers. By deleting the latter two obvious cases of contradiction to the experimental results, naturally, the periodically grating assembly mechanism can be proven to be the only feasible mechanism. In summary, if investigations were conducted only by characterizing the top surfaces of crystallized polymer film specimens, as conventionally done in the long past, then one would see only the patterns of rings and lamellae on top surface but miss the most critical pieces of bulk evidence hidden in the submerged interiors under the top surface. The ridge bands on the top surfaces actually correspond to regions where the interior tangential-oriented lamellae emerge to the top surfaces; by contrast, the valley bands on the top surfaces correspond to zones where radial-oriented lamellae evolving or bending from the tangential-oriented ones. Oppositely, these discussed results collectively show that either 2D or 3D continuous screw-like helices from a nucleus center inevitably result in contradictory cases of nonsynchronized pace in thick films (20 μm), as the helix-twist lamellae, bound in a common center, cannot be physically aligned in perfect pitch pace in 2D or 3D space as they extend outward [30]

**Figure 9:**
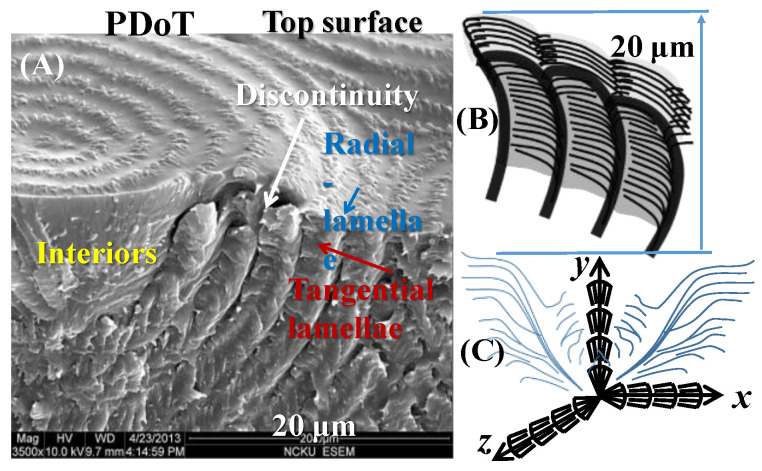
3D crystal assemblies in PDoT (m = 12) aggregates: (**A**) SEM revealing a multi-shell interior responsible for blue–orange birefringence alternate rings: (**B**) 2D cross-hatch grating assembly; (**C**) cross-hatch (corrugate-board) stacking from a common nucleus center in 3D assembly leading inevitably to onion-shell-like structures in thick films. (Figure 9B,C Reprinted with permission from ref. [30]. Copyright 2018 Royal Society of Chemistry).

In the interior of the dual-birefringence ring-banded PDT spherulites (Figure 9), it is clear that no single-crystalline lamellae continuously helix-twist such as the double-helix conformation of DNA macromolecules from a common nucleation center are seen anywhere in the banded PDT spherulites. Instead, each of the hemispheroid shells in the banded PDT spherulites is bordered with discontinuous tangential/radial interfaces, where the interior lamellae mutually intersect like a cross-hatch grating. Note that some of the tangential-oriented lamellae may either branch out or occasionally twist by ca. 90° angles to merge with the radial lamellae. If investigators had focused their analyses only on the top surfaces of the crystallized polymer films but overlooked the lamellae assembly in the interiors or how the top-surface morphology correlates with interior lamellae, it would be easy to confuse the occasional branching/bending/twisting on the top surfaces with lamellae undergoing 360° continuous helices.

**Figure 10:**
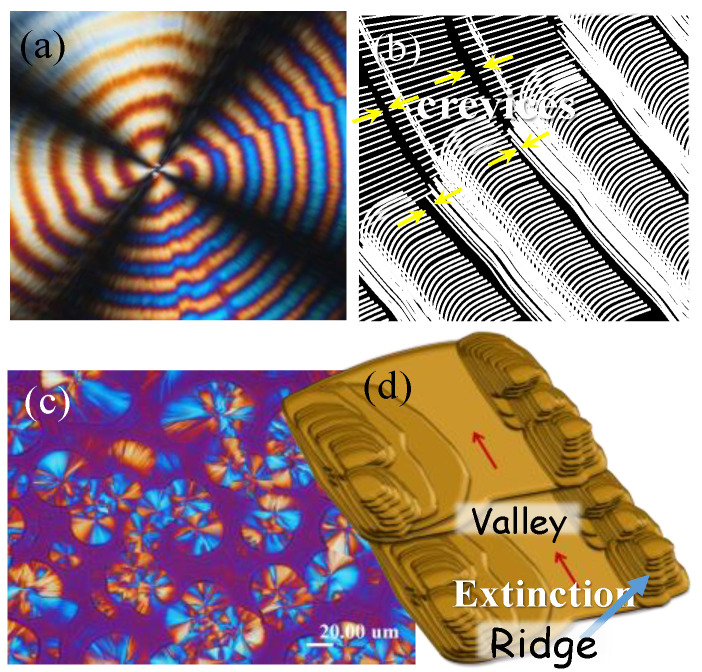
Crystal grating-assembly in double-banded vs. terrace-assembly in extinction-banded polymer spherulites: (**a**) POM micrograph; (**b**) schematic of grating architecture for dissected interior of PTT double-banded spherulites in 3~5 μm film thickness melt-crystallized at T_c_ = 165 °C, in contrast with terrace-like assembly (**c**) POM and (**d**) schematic for extinction-banded PDT spherulites at T_c_ = 110–120 °C. (Reprinted with permission from ref. [32]. Copyright 2021 Elsevier).

The optical bands in polymer spherulites can appear as a double-birefringence ring patterns or rings with extinction. Crystal assembly mechanisms in the optical extinction bands versus double birefringence rings are inherently different, and their assembled architectures are due to the completely different crystal packing mechanisms. Figure 10a shows POM micrograph of double-banded PTT spherulites’ (POM for ring patterns as inset) dissected interior (3~5 μm film thickness melt-crystallized at 165 °C), with schematics in Figure 10b revealing the interfaces and crevices between successive bands. The Interfacial discontinuity, as proved by the narrow crevices between the bands, clearly support that lamellae in the double-banded PTT were not continuously helix-twist. The interior lamellae and banded PTT were assembled as periodic gratings, with twist occurring in the interfacial boundary, signaling a discontinuity. It is worth comparing the assembly in the double-banded PTT to the dramatically different morphology of extinction-banded morphology, as represented by PDT crystallized at T_c_ = 120 °C, as shown in Figure 10c,d. The epicycloid extinction-banded PDT spherulites crystallized at high T_c_ (>110 °C) were composed of terrace-like single crystals packed along the circumferential direction of the ridge band, whereas the extinction region was due to the periodic growth precipitation [27]. The evidence in this comparative study has collectively reached an advancement of the 3D depiction of PDT dual-birefringence-banded spherulites, which differs significantly from extinction-banded ones.

**Figure 11:**
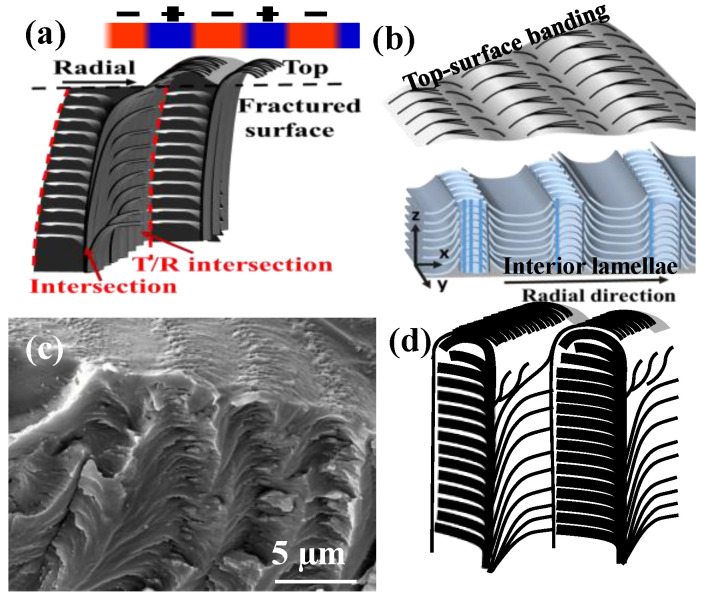
Schemes for SEM-analyzed morphology details in (**a**) lateral view of lamellae, (**b**) top and interior (Reprinted with permission form ref. [34]. Copyright 2021 John Wiley and Sons), (**c**) SEM micrograph, and (**d**) top-surface bands in correlation with interior assembly of ring-banded PDoT spherulites melt crystallized at T_c_ = 96 °C. (Figure 11b,d Reprinted with permission from ref. [30]. Copyright 2018 Royal Society of Chemistry).

Figure 11a,b show the general schemes depicting the interior and top-surface vs. interior, respectively, of the universal grating assembly in the periodically assembled crystals. Figure 11c is the SEM micrograph for PDoT revealing both the top-surface bands and interior lamellae directly underneath these periodic bands. Figure 11d shows an enlarged scheme of the fractured interior along the radial direction on the top surface, as well as the interior. The dimensions of the interior radial-oriented lamellae approximately matches with that of the ridge band on the top surface. The exact measures of these two dimensions may differ slightly, which is due to the fact that as the interior tangential lamellae emerge upward to the top surface, they have to bend downward to become radial-oriented lamellae. By contrast, the interior radial lamellae simply branch out horizontally till impinging with the next tangential layer. Thus, the interior radial lamellae tend to be slightly longer (2.6–2.8 μm) than the top-surface radial-oriented lamellae on the ridge band (ca. 2.1 μm). Note that the tangential layer was ca. 1.5–2.0 μm; thus, the total thickness of radial+tangential shells would be roughly 4–5 μm, which is the cross-bar pitch of the corrugate-board structure in PDoT at T_c_ = 96 °C.

**Figure 12:**
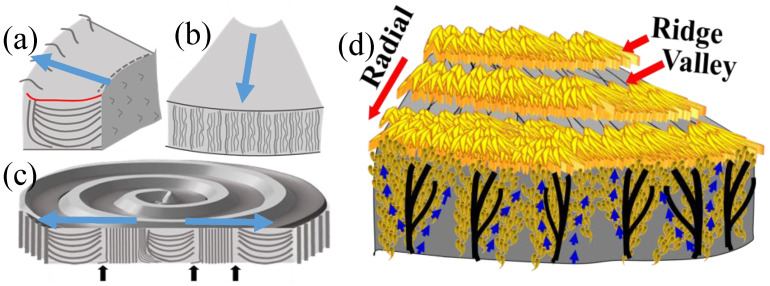
General periodic assembly mechanism for top-surface bands and interior cross-hatch lamellae: (**a**) block of stacked lamellae from valley to ridge cut in radial direction; (**b**) block of stacked lamellae from valley to ridge cut in tangential direction; (**c**) top-surface bands and interior lamellae cut in lateral view; (**d**) branched patterns made of crystal-by-crystal assembly into periodic rings on top and cross-hatch lamellae in interior.

Further dissection–morphology details are justified in constructing the general periodic assembly mechanism for top-surface bands and interior cross-hatch lamellae. The main features in the cyclic growth are that branches are inevitable in growing to fill an ever-expanding space (increasing with respect to cubic radius in 3D or squared radius in 2D) from a tiny nucleus sheaf to final fully grown spherical entity. Figure 12a–c shows the cut blocks of the lamellar stacks in one cycle from ridge to valley, exposing (a) the lateral side of the stacked lamellae, (b) circumferential side of stacked lamellae, and (c) top and lateral views of banded spherulites. It would be erroneous if one sees that the interior lamellae are monotonous single stalks and continuous in growing from nucleus to periphery of spherulites. Branching is a universal feature, as the lamellae self-assemble to fill the aggregated spherulites. Figure 12d shows that the branches are made of crystal-by-crystal assembly both on the top surface and interior. “Brick-by-brick” (single crystal-by-crystal) assembly means that the lamellae are not continuous and there actually is discontinuity in crystal boundaries when final periodic aggregates are formed.

### 3.5. Periodic Microstructures as Functional Photonic Crystals

**Figure 13:**
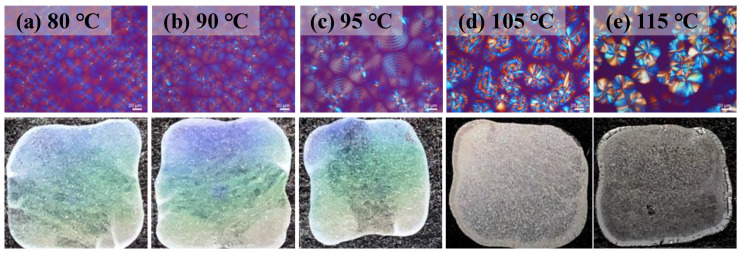
POM images (upper row, scale bar = 20 μm) and photonic iridescence (lower row) of neat PDT crystallized at different crystallized temperature (T_c_): (**a**) 80, (**b**) 90, (**c**) 95, (**d**) 105 and (**e**) 115 °C after melting at T_max_ = 165 °C for 1 min, showing the correlation of the periodic bands and qualitative iridescence intensity. (Square box dimension in iridescence = 1.8 cm× 1.8 cm).

Among the series of arylate polyesters, poly(decamethylene terephthalate) (PDT) crystals are taken as a handy example for illustrating the correlation of a periodically assembled microstructure serving as structural crystals for light interference. All PDT spherulites were typically circular in shape and exhibited distinct birefringence rings. The temperature-dependent periodic morphologies of the PDT showed a systematic trend of variation at T_c_ = 80–120 °C. Iridescence experiments on the PDT crystallized at these Tc were performed in accordance with the procedures and setup for white light interference as documented in the relevant literature [34,35,36]. Figure 13a–e show POM images (upper row) and iridescence photo shots (lower row), where the iridescence spectra generally took a softer hue color due to the lesser order of the assembly in the crystallized neat PDT films. In the range Tc = 80 ℃ to 95 ℃, the crystallized PDT samples had the dimensions of band-ring spacing, varying from ~2.7 μm to 3.29 μm, and a spherulites radius ranging ~16.4 μm. The lesser order of the rings and smaller size of the spherulites both unfavorably affected the formation of the color, causing it to appear as a softer shade of color. In comparison, the morphology of the PDT could not produce color in the transitional morphology and epicycloid extinction ring-banded morphology. Transitional morphology is characterized by an irregular arrangement of ridges and valleys devoid of the periodic fractal branching of ridges and valleys crystal. By contrast, the fully extinction-banded PDT ridge is entirely packed with terrace-like single-crystal flat plates and the amorphous ingredient accumulates on the valley. These two situations are incapable of causing light interference because the grating architecture’s condition is unconfirmed. The inter-bands spacing increased from 80 °C until Tc = 95 ℃; with the crystallization temperature at 105 °C or higher, the morphology changes from the regular ring-banded pattern to another transitional morphology, resulting in few nuclei. This indicates that crystallization kinetics strongly governs the formation of ring bands. When the Tc increased above 115 °C, the ring-banded morphology changed to epicycloid extinction ring banded. Only the ordered structure of the ring-banded morphology crystallized between Tc = 80 °C and 95 °C supports effective interference with white light to produce noticeable iridescence spectra.

## 4. Conclusions

The birefringent-banded aggregates of PDT in comparison with PTT and PDoT display optically alternating blue/orange rings, which are composed of cross-hatch gratings of multi-shells, with each shell taking a corrugate structure composed of lamellae perpendicularly intersecting each other. The interior dissection results for the birefringent-banded PDT spherulites revealed very critical pieces of evidence for the realistic mechanisms of lamellae starting from nano- to micro-assembly and finally to aggregated crystal entities with alternate birefringent bands, which can be perfectly correlated to the top-surface and interior crystal architectures in dimensions and orientations. The radial lamellae were roughly 90° angle perpendicularly intersecting with the tangential ones; therefore, the top-surface valley band region appeared to be a submerged “U-shape”, where the interior radial lamellae were located directly underneath. The total layer thickness (4–5 µm) of the ridge and valley bands, as revealed in the SEM results, equaled the inter-band spacing in the POM results.

Thus, by the results summed up from analyses on several polymers with periodic assemblies, it can be concluded that the periodicity, as revealed in optical patterns and microscopy dissected interiors, are based on discontinuous grating-like lamellar structures rather than continuous screw-like helices. The former is based on crystal-by-crystal self-assembly with periodic branching and discontinuous intersection, which matches with the nature of the formation and assembly from nanocrystals to polycrystalline architectures with periodicity in optical patterns and morphology. By examining the nano- to micro-assembly in hierarchical structures to periodic patterns in crystal aggregates of arylates, this work has provided clear and strong evidence of the absence of continuity or helical geometry. The lamellae in two types of banded PDT spherulites (extinction vs. birefringence types) display diversified mechanisms of banding periodicity that both are different but collectively cannot be described by the classical models of continuous lamellar helix-twisting. The interior lamellae dissection and analysis on distinctly different mechanisms of lamellar assembly in the extinction vs. birefringence-banded PDT spherulites, reinforced by analyses based on other arylate polyesters, provide deeper understanding of mechanisms of periodic crystal assembly phenomena that have long intrigued the science community.

## Data Availability

The data presented in this study are available on request from the corresponding author.

## Supplementary Materials

The following supporting information can be downloaded at: https://www.mdpi.com/article/10.3390/nano13061016/s1, Figure S1. Double-birefringence ring bands in PDT spherulites of increasing film thickness: (a) 300–500 nm by spin-casting, (b) 3–5 um, (c) 7–10 um, (d) 15–20 um, all crystallized at T_c_ = 90 °C; Figure S2. POM micrographs for birefringent-banded spherulites of neat PDT melt-crystallized at T_c_ = 95 °C with specimens held at T_max_ (165 °C) for different times (t_max_): (a) 1 min, (b) 15 min, (c) 30 min, (d) 60 min, (e) 90 min, and (f) 120 min. (All film thickness kept at ca. 2–3 µm).
