# Supplementary material for: From Nano-Crystals to Periodically Aggregated Assembly in Arylate Polyesters—Continuous Helicoid or Discrete Cross-Hatch Grating?
Source: Nanomaterials (Basel). 2023 Mar 11;13(6):1016. doi: 10.3390/nano13061016 (PMC10052005; doi:10.3390/nano13061016)
Supplement: Supplementary file 1 [file nanomaterials-13-01016-s001.zip › nanomaterials-2255548-supplementary.pdf]

nanomaterials

Article

From Nano-Crystals to Periodically Aggregated Assembly in Arylate Polyesters—Continuous Helicoid or Discrete Cross-Hatch Grating?

Cheng-En Yang [1], Selvaraj Nagarajan [1], Widyantari Rahmayanti [1], Chean-Cheng Su [2] and Eamor M. Woo [1,*]

[1] Department of Chemical Engineering, National Cheng Kung University, No. 1, University Road, Tainan 701-01, Taiwan
[2] Department of Chemical and Materials Engineering, National University of Kaohsiung, No. 700, Kaohsiung University Rd., Nan-Tzu Dist., Kaohsiung 811-48, Taiwan
* Correspondence: emwoo@mail.ncku.edu.tw; Tel.: +886-2757575 (ext. 62670)

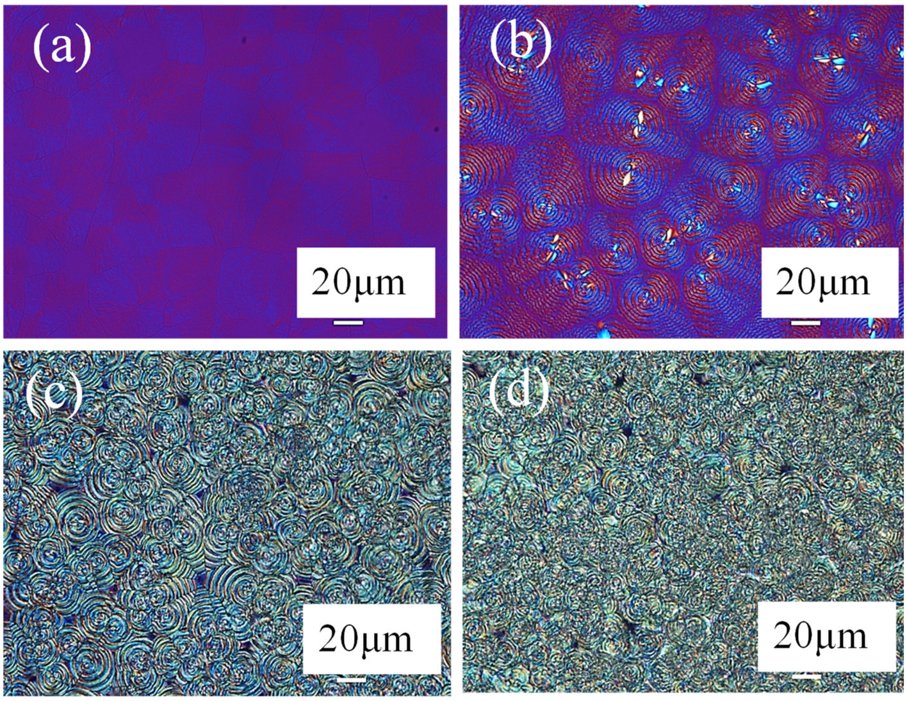

Figure S1. Double-birefringence ring bands in PDT spherulites of increasing film thickness: (**a**) 300–500 nm by spin-casting, (**b**) 3–5 um, (**c**) 7–10 um, (**d**) 15–20 um, all crystallized at T_c = 90 °C.

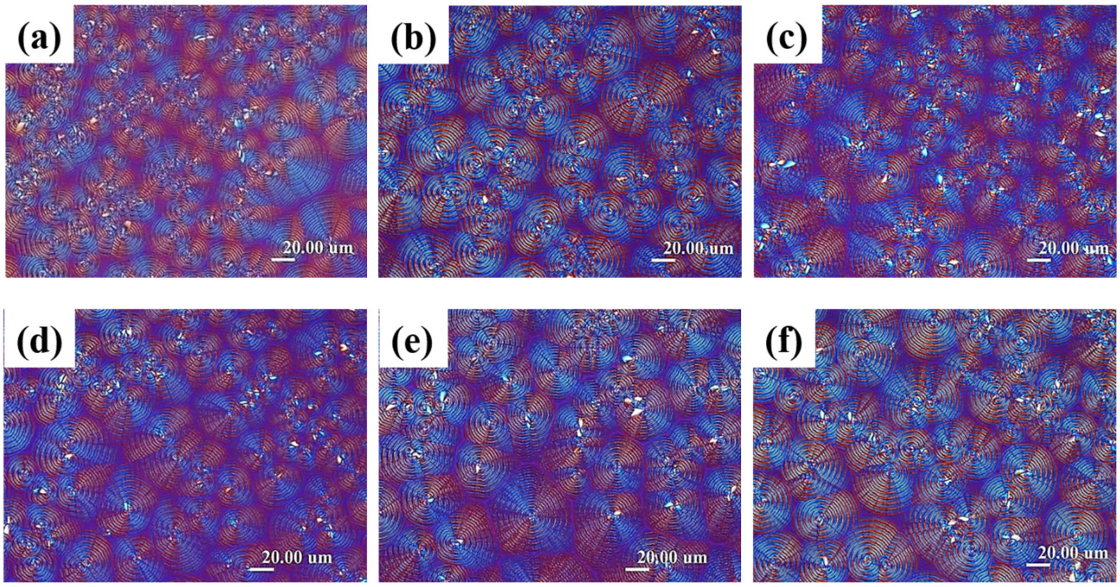

Figure S2. POM micrographs for birefringent-banded spherulites of neat PDT melt-crystallized at T_c = 95 °C with specimens held at T_{max} (165 °C) for different times (t_{max}): (**a**) 1min, (**b**) 15min, (**c**) 30min, (**d**) 60min, (**e**) 90min, and (**f**) 120 min. [All film thickness kept at ca. 2–3 μm].
